# A combination of targeted enrichment methodologies for whole-exome sequencing reveals novel pathogenic mutations

**DOI:** 10.1038/srep09331

**Published:** 2015-03-19

**Authors:** Fuyuki Miya, Mitsuhiro Kato, Tadashi Shiohama, Nobuhiko Okamoto, Shinji Saitoh, Mami Yamasaki, Daichi Shigemizu, Tetsuo Abe, Takashi Morizono, Keith A. Boroevich, Kenjiro Kosaki, Yonehiro Kanemura, Tatsuhiko Tsunoda

**Affiliations:** 1 Laboratory for Medical Science Mathematics, RIKEN Center for Integrative Medical Sciences, Yokohama, Japan; 2 Department of Pediatrics, Yamagata University Faculty of Medicine, Yamagata, Japan; 3 Department of Pediatrics, Graduate School of Medicine, Chiba University, Chiba, Japan; 4 Department of Medical Genetics, Osaka Medical Center and Research Institute for Maternal and Child Health, Osaka, Japan; 5 Department of Pediatrics and Neonatology, Nagoya City University Graduate School of Medical Sciences, Nagoya, Japan; 6 Department of Pediatric Neurosurgery, Takatsuki General Hospital, Osaka, Japan; 7 Center for Medical Genetics, Keio University School of Medicine, Tokyo, Japan; 8 Division of Regenerative Medicine, Institute for Clinical Research, Osaka National Hospital, National Hospital Organization, Osaka, Japan; 9 Department of Neurosurgery, Osaka National Hospital, National Hospital Organization, Osaka, Japan

## Abstract

Whole-exome sequencing (WES) is a useful method to identify disease-causing mutations, however, often no candidate mutations are identified using commonly available targeted probe sets. In a recent analysis, we also could not find candidate mutations for 20.9% (9/43) of our pedigrees with congenital neurological disorder using pre-designed capture probes (SureSelect V4 or V5). One possible cause for this lack of candidates is that standard WES cannot sequence all protein-coding sequences (CDS) due to capture probe design and regions of low coverage, which account for approximately 10% of all CDS regions. In this study, we combined a selective circularization-based target enrichment method (HaloPlex) with a hybrid capture method (SureSelect V5; WES), and achieved a more complete coverage of CDS regions (~97% of all CDS). We applied this approach to 7 (SureSelect V5) out of 9 pedigrees with no candidates through standard WES analysis and identified novel pathogenic mutations in one pedigree. The application of this effective combination of targeted enrichment methodologies can be expected to aid in the identification of novel pathogenic mutations previously missed by standard WES analysis.

Next-generation sequencing (NGS) technology has enabled genome sequencing in a cost-effective and high-throughput manner^1^. Whole-exome sequencing (WES) is designed to target exonic regions^2^,^3^ with the aim of identification of disease-causing mutations. More than 90% of known pathogenic mutations registered in NCBI ClinVar database are located in protein-coding DNA sequence (CDS) regions (as of Apr. 3, 2014). However, the reported success rate of WES for Mendelian diseases^4^,^5^ ranges from 20% to 50%^5^,^6^. One possible cause for the low success rate is that current WES does not sequence all CDS due to capture probe design and regions of low coverage (<10×).

Current techniques for targeted enrichment can be categorized into three methods^7^,^8^: ‘hybrid capture' (e.g. Agilent SureSelect), ‘selective circularization' (e.g. Agilent HaloPlex) and ‘PCR amplification' (e.g. RainDance Technologies). The hybrid capture method is the most commonly used for WES analysis because this method can handle large target regions. However, this hybrid capture method has probe design challenges for capturing repetitive and guanine-cytosine (GC) rich regions^9^. While the selective circularization and PCR amplification methods can better target these difficult regions (Supplementary Fig. S1), these methods can only handle smaller target regions (~<5 Mb). Therefore, a combination of the different methods should enable higher coverage of the CDS regions, increasing the success rate of WES analysis for mutations detection.

We recently established a consortium with the aim of identifying disease-causing mutations and applying that knowledge to clinical diagnosis of congenital neurological diseases (microcephaly, cortical dysplasia, hydrocephalus, agenesis of corpus callosum, cerebellar hypoplasia, macrocephaly and others) with a group of research institutes and hospitals in Japan, and to that end, performed WES analysis (hybrid capture method) on several families. However, no candidate mutations were observed in a subset of the analyzed families. Here we complemented hybrid capture WES with the selective circularization method enabling us to greatly increase our coverage of CDS regions. This approach was applied to several families in which no candidates were found using WES analysis and we successfully identified novel pathogenic mutation in one such family. This effective approach of combining targeted enrichment technologies can be expected to aid in the identification of novel pathogenic mutations for samples where standard WES analysis has previously failed.

## Results

### Coverage of CDS with WES

The Agilent SureSelect V4 and V5 platforms used in our WES analysis are designed to cover 93.9% and 96.5% of all CDS regions (NCBI GRCh37.p10; 33.8 Mb), respectively. The observed coverage of all CDS regions with a read depth ≥10 were 90.6% and 93.9% on average for SureSelect V4 and V5, respectively (Table 1 and Supplementary Table S1), similar or higher than those reported in previous studies^8^,^9^,^10^. Note that regions with a read depth ≥10 (RD10) are subject to variant calling in our pipeline (see Methods) and therefore used to define regions that are sufficiently sequenced.

We investigated the coverage of CDS regions for additional platforms, NimbleGen SeqCap v2.0 (SeqCap) and Illumina TruSeq Exome Enrichment Kit (TruSeq), which were designed to target 96.1% and 94.3% of all CDS regions, and had an observed RD10 coverage of all CDS regions of 93.5% and 90.2%, respectively. Consequently, our investigation of the hybrid capture method kits, which are more commonly used for WES analysis, revealed that they could not sequence approximately 7% to 10% of all CDS regions (Table 1).

### Success rate of WES analysis

In our previous study, we performed WES on 50 affected and 88 unaffected individuals of congenital neurological disease in 43 pedigrees (n = 138 exomes). We identified known pathogenic genes mutations in 13 pedigrees (success rate: 13/43 = 30.2%) and one or more candidate mutations in 21 pedigrees (48.8%). However, no candidate mutations were detected in 9 of the pedigrees (20.9%, see Supplementary Table S2 for each experimental platform). Since the causative mutations of these individuals may be located in the unsequenced CDS regions, we designed custom probes to sequence an additional 7 to 10% of all CDS regions not sequenced by WES, in an attempt to identify new candidate mutations.

### Design of complementary custom CDS sequencing (CCCS)

As described above, one of the possible causes of no candidates is WES does not sequence all CDS regions due to capture probe design and regions of low coverage (<10×). To overcome this limitation, we designed HaloPlex (Agilent) custom molecular inversion probes for the CDS regions not sequenced by WES. The design of probes for complementary custom CDS sequencing (CCCS) was conducted based on the WES data of the Japanese individual NA18943 with SureSelect V5. The 2,171,214 bases of CDS with a read depth <15 were selected and 181,915 probes were designed by SureDesign software (Agilent) when allowing for the design of short amplicons. Of these, 120,383 probes satisfied our selection criteria and were used for CCCS (see the details in Methods and Supplementary Table S3). The probe set for CCCS was predicted to cover 9.8% of all CDS regions (3.3 Mb), 67.8% of which were of CDS regions with a read depth <15 or not covered by probes in WES (1,472,963 out of 2,171,214 bases: 4.4% of all CDS regions).

### Coverage of CDS with CCCS

**Figure 1:**
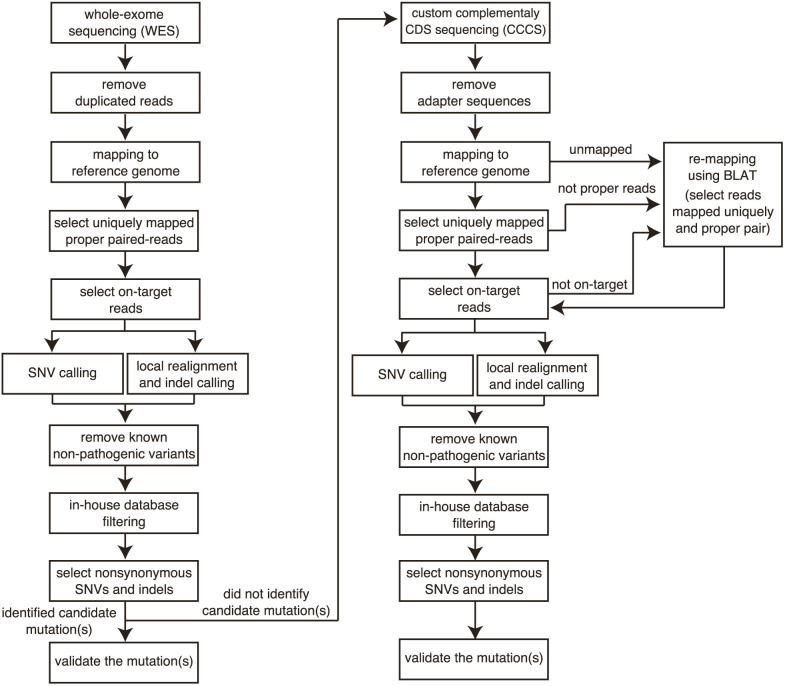
Analysis pipeline for combination of WES and CCCS. WES was performed for all pedigrees. Complementary CDS sequencing (CCCS) was then performed in those where no candidate mutations were identified. The three steps, PCR duplication filter, adapter sequence removal and remapping using BLAT, differ between WES and CCCS analysis.

**Figure 2:**
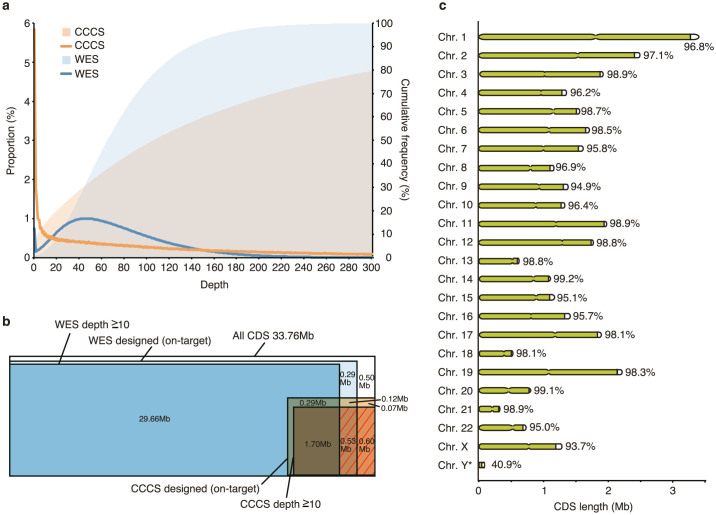
Sequencing of CDS regions. (a) Read depth distribution of on-target CDS regions in WES and CCCS. The blue and orange lines indicate the proportion and the filled areas indicate cumulative frequency. (b) CDS coverage by WES and CCCS. The red shaded area indicates the bases sequenced only with CCCS. (c) Combined sequencing coverage for WES and CCCS by chromosome. Bar lengths do not reflect chromosome length but the total number of CDS bases. Lime green and percentage indicate proportion of RD10 bases for each chromosome. All values are average across the 28 tested samples.

No candidate mutations were detected in 9 pedigrees: two Agilent SureSelect V4 and seven Agilent SureSelect V5 WES analyses. Since the probe set for CCCS was designed based on the unsequenced regions of SureSelect V5 data, we performed CCCS for the 27 samples in only the 7 SureSelect V5 pedigrees and one additional sample (NA18943), which was used to design the CCCS probes (n = 28 in total, Fig. 1). An average of 1.74 Gb of sequence was generated from CCCS, of which, 0.97 Gb mapped uniquely to the reference genome as proper-paired reads within on-target regions by the short read mapping algorithm BWA. For unmapped and improperly paired reads, we performed remapping using BLAT (Fig. 1). In total, an average of 1.07 Gb of read sequences per sample (increase of 106 Mb by BLAT; ~11%) was at sufficient coverage for variant calling. The average read depth of CCCS targeted regions without and with the BLAT alignment was 169.9 and 194.6, respectively (Fig. 2a and Supplementary Table S4). CCCS targeted regions with an RD10 accounted for 8.4% of all CDS regions on average (2.83 Mb).

### Coverage of CDS with combination of WES and CCCS

The SureSelect V5 (WES) and CCCS were designed to cover 96.5% and 9.8% of all CDS regions, respectively. The combination of WES and CCCS was designed to cover 98.5% of all CDS regions, as some of the designed probes overlap between the two methods. The observed average RD10 coverage of the CDS regions was 93.7% for WES and 97.1% for the combination method in the 28 samples (Fig. 2b and Supplementary Table S5). The percentage of RD10 CDS regions was similar across the 28 samples and the standard deviation for WES and combination method was 0.37% and 0.15%, respectively (Supplementary Table S5). We further investigated read depths of regions across all 28 samples which had a read depth <15 in NA18943 sample (used for CCCS probe design) and covered by CCCS probes, except for those on sex chromosomes. The total number of bases examined was 1,368,357. The mean depths of the regions were 4.1 and 268.2 in WES and CCCS, respectively (Supplementary Fig. 2). The Spearman's rank-correlation coefficients of the depths for all the bases between 28 samples (378 combinations) were 0.920 ± 0.005 (mean ± SD) and 0.928 ± 0.032 (mean ± SD) in WES and CCCS, respectively (Supplementary Fig. 2). In addition, we investigated the read depth of all bases in targeted CDS regions in the WES, except for those on sex chromosomes (311,295,571 bases). The mean depth and Spearman's rank-correlation coefficients were 75.1 and 0.946 ± 0.003 (mean ± SD), respectively. These results indicate that the CDS regions not sequenced by WES and sequenced by CCCS were remarkably similar in all samples, demonstrating that the single CCCS design is broadly useful across samples. We also calculated the percentage of CDS regions with an RD10 for each chromosome (Fig. 2c and Supplementary Table S6). Most of chromosomes showed high CDS coverage (more than 98%), while sex chromosomes had low coverage, in particular, the Y chromosome (40.9% coverage).

### Evaluation of variant calls on WES and CCCS data

We performed variant calling based on in-house calling algorithm (Methods) on the WES and CCCS data. To evaluate the accuracy of our calling algorithm, we compared the results to array-based genotype calls using the Illumina HumanOmniExpressExome SNP array for the 7 samples. The number of SNVs available for verification was 243,573 from WES and 26,168 from CCCS. Respectively, the false positive and false negative rates were estimated to be 0.021% and 0.064% for WES and 0.038% and 0.27% for CCCS using a conservative estimation (see the details in Supplementary Data 1 and Supplementary Table S7–S11). We also performed the same comparisons for SNVs with read sequences rescued by BLAT mapping. No false positive or false negatives were observed (0/260). In this study, although we used combination of ‘aln/sampe' BWA option and BLAT, the ‘mem' option of BWA may be an equally useful method (Supplementary Table S12).

### Identification of novel compound heterozygous *ASPM* mutation in microcephaly family

**Figure 3:**
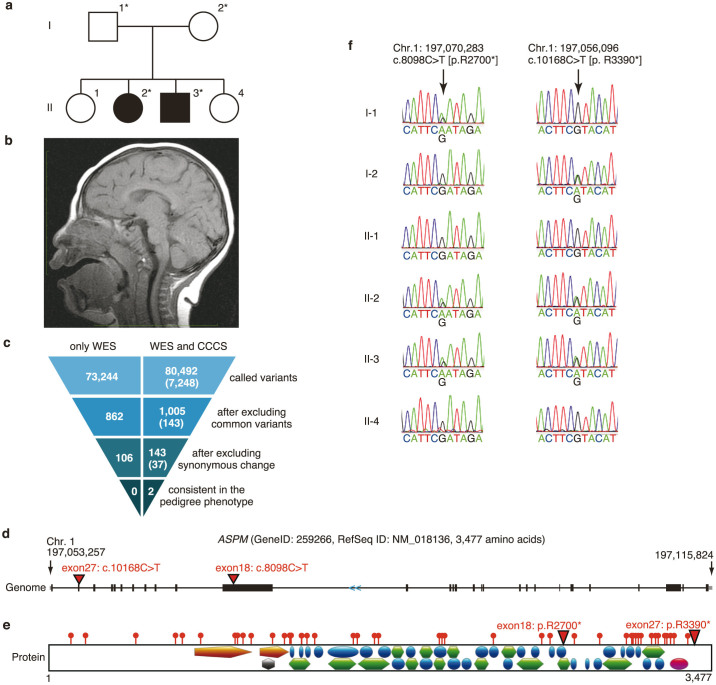
Identified mutation in a family with microcephaly. (a) Family tree of the pedigree with microcephaly. Shaded symbols denote affected individuals. Asterisks denote NGS was performed. (b) Sagittal T1-weighted brain magnetic resonance image (MRI) of the II-2 individual at 4 years of age shows frontal sloping and reduced volume of the brain, particularly the frontal lobe of the cerebrum. (c) Filtering the candidate mutations for the II-2 individual. The numbers in parenthesis represent the number of called variants with CCCS. Overlapping variants between WES and CCCS are not excluded. The other individuals are shown in Supplementary Fig. S3. The top row shows the variant counts called by ‘WES' and ‘WES and CCCS'. The second row shows counts after excluding known variants found in databases, except for known pathogenic mutations. The third row shows variant counts after excluding synonymous changes. Finally, the last raw of variant counts is consistent with the phenotype in the pedigree (i.e., total number of the autosomal recessive and compound heterozygous variants). (d) *ASPM* gene in human genome. Gray and black box indicate exonic untranslated regions (UTR) and CDS regions respectively. Red triangles indicate loci of identified mutation. Blue arrow (<<) indicates the coding direction. (e) Domains and mutations in the ASPM protein. Red pins indicate loci of known nonsense mutations in HGMD and ClinVar databases at 2014 June. Red triangles indicate loci of identified mutation. Orange pentagon, black hexagon, green hexagon and magenta oval denote calponin homology (IPR001715: InterPro ID), calmodulin-regulated spectrin-associated protein CH (IPR022613), P-loop containing nuclease triphosphate hydrolase (IPR027417) and armadillo-type hold domain (IPR016024), respectively. Blue oval denotes IQ motif, EF-hand binding site (IPR0000048). (f) Sanger sequencing data of the identified mutation.

We performed a combination of WES and CCCS on 27 samples (7 pedigrees), and identified a novel compound heterozygote of *ASPM* (RefSeqID: NM_018136.4, also known as *MCPH5*) mutation in one microcephaly family (Supplementary Data 2). This mutation was composed of two nonsense variants, c.8098C>T [p.R2700*] and c.10168C>T [p.R3390*], identified through WES and CCCS analysis, respectively (Fig. 3 and Supplementary Fig. S3). The variant call of latter locus was missed in WES as the region had low read depth (<10) in all samples. The variants were validated for all six individuals in the family using Sanger sequencing. The parents were carriers of one variant each and the unaffected children showed no mutations (Fig. 3f). These variants were absent from our in-house and public databases: dbSNP, 1000 Genomes Project, NHLBI Exome Sequencing Project (ESP6500), Human Genetic Variation Database (HGVD), NCBI ClinVar and Human Gene Mutation Database (HGMD).

The *ASPM* is a known disease-causing gene of microcephaly (autosomal recessive primary microcecphalay-5, MCPH5; OMIM #608716) and plays a key role in proper neurogenesis and neuronal migration^11^, cerebral cortex size^12^ and mitotic spindle function^12^,^13^,^14^. Previous studies have reported that phenotypic variation in head circumference and mental retardation was not correlated to the position of the mutation in *ASPM*^15^,^16^. Mutations on C-terminal regions of the protein (e.g. p.Y3353*^16^ and p.R3354*^17^) have also been reported as pathogenic mutations (Fig. 3e).

## Discussion

We demonstrated that the combination of different targeted enrichment methods, WES with a hybrid capture method and CCCS with a selective circularization method, can successfully identify novel pathogenic mutation in subjects where WES alone has previously failed. We applied the combination method to 7 pedigrees and, in one of them, a pathogenic mutation was identified. Overall, we identified pathogenic or candidate mutations in 35 out of 43 pedigrees (35/43 = 81.4%). Although the success rate depends on multiple factors, such as the disorder, inheritance pattern, sample size of pedigree, this combination method can contribute to an increase in success rate of exome analysis. When no candidate mutations are identified with standard WES, one option may be to consider CCCS analysis.

The combination method predicted 98.6% coverage of CDS regions by probe design and produced an observed RD10 for 97.4% of all CDS regions. The average increase of regions with an RD10 was 4–7%. When exploring causative mutations of Mendelian diseases, it is important to sequence as much of the CDS region as possible as most mutations exist in the CDS. Applying additional WES (using SureSelect V5) is expected to result in only a small increase in the CDS coverage. However, a combination of the different methods (WES + HaloPlex) enables higher coverage of the CDS regions, because the HaloPlex can sequence the CDS regions that could not be captured in the single WES platform. An alternative approach to CCCS may be to sequence the CDS regions not covered by WES using Sanger sequencing, but this is impractical since the regions are larger than 2 Mb. Using multiple pre-designed WES platforms is another option that increase the CDS coverage as the designed regions differ between each platform^9^. However, the cost of such an approach approximately doubles for each platform added. In contrast, our CCCS method is cost-effective (~$300/sample) since the targeted size is limited.

The average RD10 coverage of CDS regions varied among chromosomes. While an RD10 was observed for more than 98% of CDS regions in half of the chromosomes, that of Y chromosome was particularly low (40.9%, Supplementary Table S6). This is likely due to pseudoautosomal regions (PAR) on the Y chromosome, high sequence similarity to X chromosome, and many repeat sequences in male specific regions of Y chromosome (MSY)^18^. If the criteria for probe designing and read mapping are made less restrictive, the coverage of Y chromosome may slightly increase. There is a trade-off between coverage of CDS and accuracy of the genotype calls.

We compared the read depths across all samples for the WES and CCCS data using the Spearman's correlation coefficients and the Pearson's correlation coefficients. High correlation coefficients were obtained in both statistical approaches, although the Pearson's correlation coefficients were slightly lower, particularly in low depth regions, than Spearman's correlation coefficients (Supplementary Fig. S2). The reasons for this difference are likely because, 1) the distribution of read depths deviates from normal distribution because many bases had zero depth, and 2) the read depths are quite different across samples in the regions where the average of read depths are low. These results also imply that the design of additional probe sets using only a single sample, such as in our CCCS, is sufficient as long as the same WES platform are used.

Finally, we considered why no candidate mutations were observed in the remaining six pedigrees. Possible reasons are that the mutations could be synonymous variant in exonic regions^19^, located in non-targeted intronic, intergenic, unannotated genic, or untranslated regions (UTR), or the penetrance of the disease could be low (the number of synonymous CDS and total UTR variants, after filtering, for the six pedigrees is in Supplementary Table S13. It should be noted that SureSelect V5 probes covered only 14.8% of all UTRs). Currently, it is a great challenge to consider not only synonymous variants but also this issue of low penetrance. Gene-based association studies (e.g. SKAT^20^,^21^,^22^) would be a useful solution, although a large number of samples composed of affected and unaffected individuals, such as for GWAS^23^,^24^, is necessary. Another possibility could be that the causative mutations are located in remaining exonic regions that were not sequenced. Long-read sequencing by a 3rd or later generation sequencer^25^ will enable researchers to target these regions in the near future.

In conclusion, our combination method of WES and CCCS contributed to an increase in the coverage of CDS (97.4%) and was successfully able to identify novel pathogenic mutation. We believe that this combination method will contribute to the identification of novel causative mutations, which are inaccessible by current pre-designed standard WES analysis, and to a greater understanding of mechanisms and therapies for diseases.

## Methods

### Subjects

With approval of the ethics committee of RIKEN, Yamagata University Faculty of Medicine, Chiba University, Osaka Medical Center and Research Institute for Maternal and Child Health, Nagoya City University Graduate School of Medical Sciences, Takatsuki General Hospital and Osaka National Hospital, this research was performed. The methods were carried out in accordance with the approved guidelines. Written informed consent was obtained from all participants. The genomic DNA of each subject was extracted from peripheral blood. The quality of our SNV calling and the coverage were tested on an EBV-transformed lymphoblastoid cell line (LCL) derived from a HapMap-JPT NA18943 male obtained from Coriell as previously reported^26^. After culturing the cells, the genomic DNA was extracted using DNeasy Blood & Tissue Kit (Qiagen).

### Whole-exome sequencing

Following DNA extraction, 3 μg of each sample DNA was sheared to 150–200 bp using the Covaris DNA Shearing System. To capture the exonic DNA, we used the SureSelectXT Human All Exon V4 or V5 capture library (Agilent) for 50 Mb of exonic regions. The sequence library was constructed with the SureSelect XT Target Enrichment System for Illumina Paired-End Sequencing Library kit (Agilent) according to the manufacturer's instructions. We performed DNA sequencing of 100 or 101-bp paired-end reads using the Illumina HiSeq 2000 sequencer.

### SNV and indel calling of WES

We performed SNV and indel calling for the WES data as previously reported^26^,^27^. Briefly, we aligned the WES reads to the Human reference genome (GRCh37/hg19), excluded PCR duplicated reads, and extracted uniquely mapped and properly paired reads with an insert size within ±2SD of the mean. Variant calling was performed in on-target regions within 100 bp upstream and downstream of the designed capture probes.

### Comparison with other platforms

Probe information of SeqCap and TruSeq were obtained from each product company web site. WES data for the platforms were obtained from NCBI SRA (Sequence Read Archive), SRX083312 and SRX083313^9^. The WES data was analyzed and SNVs were called as described above. The RD10 coverage of the CDS regions was calculated using BEDtools (ver.2.13.3) and in-house programs.

### Custom complement CDS sequencing (CCCS)

The design of probes for CCCS was based on the WES data for the NA18943 Japanese individual from SureSelect V5. We designed custom HaloPlex probes using the SureDesign software (Agilent) for the exonic CDS regions with a read depth <15 in the SureSelect V5 analysis. First, the probes were designed using the “Maximize Specificity” parameter on the SureDesign software. We then performed BLAT (ver.35) alignment for simulated 100 bp paired-end reads based on the designed probes, and accepted only the probes with paired-end reads uniquely mapped to reference genomes with an insert size less than 1,000 bp. For rejected targeted bases, we redesigned probes using both “Maximize Specificity” and “Optimize for Fragmented Samples” (permit designing short amplicon) parameters on SureDesign. In a similar way, we performed BLAT alignment and determined if the probes are acceptable. If the targeted bases were again rejected, we used “Maximize Coverage” and “Optimize for Fragmented Samples” parameters on SureDesign, and checked again using the BLAT alignment. The details for each parameter are described on the SureDesign website. The design of probes for CCCS and the sequencing were conducted using “the HaloPlex Target Enrichment System For Illumina Sequencing Kit” (Agilent) and “the Illumina HiSeq 2000 sequencer” according to the manufacturer's instructions.

### SNV and indel calling of CCCS

We removed adapter sequences from the FASTQ files of CCCS using Cutadapt (ver.1.3) as some of CCCS short amplicons included adapter sequences. The commands for Cutadapt are “cutadapt –overlap = 10 -m 50 -a AGATCGGAAGAGCACACGTCTGAACTCCAG -o output.fastq input.fastq” for forward reads and “cutadapt –overlap = 10 -m 50 -a AGATCGGAAGAGCGTCGTGTAGGGAAAGAG -o output.fastq input.fastq” for reverse reads. The sequenced reads were then mapped to the Human reference genome (GRCh37/hg19) using the Burrows-Wheeler Aligner (BWA, ver.0.6.1). Note that we did not exclude PCR duplicates for CCCS analysis. Paired reads were mapped by considering paired reads insert size (≤1,000 bp), mapping uniqueness and orientation. Since the terminal 5 bp of the sequenced reads on each end include recognition sites of restriction enzymes due to design of probes using selective circularization method, variant calling was performed in on-target regions excluding these base-pairs.

For paired reads not used for variant callings (unmapped reads, improper paired reads, mapped reads in off-target), we performed BLAT alignment and rescued additional uniquely mapped proper paired-reads. BLAT was performed with default settings (allowing up to 5 mismatched bases in each read). Read pairs that were mapped in proper orientation with a predicted insert size of 1,000 bp or less were collected and the total alignment mismatch count was calculated for each mapping location. Only read pairs that had a single mapping location for the minimum mismatch count for that pair were considered unique and were retained. SNV and insertion and deletion (indel) calling was performed using the SAMtools (ver.0.1.16) and the GATK (ver.1.6) software. The SAMtools parameters minimum base quality score was set to ≥20, maximum read depth was set to <10,000, and the others were left as the default. We then extracted the variants that differed from the reference sequence with SNP score ≥20 and consensus score ≥20, and classified the variants with an alternative allele frequency from 0.25 to 0.75 as “heterozygous SNV and/or indel” and >0.75 as “homozygous SNV and/or indel”. For indel calls, we performed local realignment of the reads overlapping the above SNVs and indels using GATK, and called the variants again. The regions with an alternative allele frequency <0.25 or read depth <10 were considered to be missing sequences. To identify candidate disease-causing mutations, we excluded known variants found in dbSNP (build138), the 1000 Genomes Project, ESP6500, HGVD and our in-house database, except for known pathogenic variants with minor allele frequency (MAF) <0.01 included in HGMD professional (ver. 2014.1, accessed on Apr. 3, 2014) or ClinVar (accessed on Apr. 3, 2014). Nongenic, intronic and synonymous variants, other than nonsynonymous variants (nonsense, missense and splice site SNVs and frame shift indels), were excluded.

### NGS base-call validation

To evaluate the accuracy of our base-calling algorithm, we compared our variant calls result with the concordant genotypes from SNP typing platform: Illumina HumanOmniExpressExome SNP array. Sanger sequence verification of variants was performed using the Applied Biosystems 3730xl DNA Analyzer. The primers sequences for accuracy check are described in Supplementary Table S8 and S11. The validation primers of the *ASPM* are ASPM_exon18F, TGCCTCTAAAAGCAGCCTGAA; ASPM_exon18R, CAGTGCGTACCCAAGCAGTTA; ASPM_exon27F, TGGTCCTTACAGGTGTTTCTGG; ASPM_exon27R, GGAGGCAGAGATTGCATTGAG.

## Author Contributions

F.M. and T.T. conceived and designed the project. M.K., N.O., S.S., M.Y., K.K. and Y.K. contributed to the original concept of the project. M.K., T.S., N.O., S.S., M.Y. and Y.K. contributed sample collection and the phenotype diagnosis. F.M. performed the experiments of WES, CCCS and Sanger sequencing, and designed the custom probes for CCCS. F.M., T.A. and T.M. analyzed the data. D.S. and K.A.B. provided the technical assistance. F.M., M.K., D.S., K.A.B. and T.T. wrote the manuscript. All authors contributed to the final manuscript.

## Supplementary Material

Supplementary Information Supplementary Information

Supplementary Information Supplementary Table S1

Supplementary Information Supplementary Table S2

Supplementary Information Supplementary Table S3

Supplementary Information Supplementary Table S4

Supplementary Information Supplementary Table S5

Supplementary Information Supplementary Table S6

Supplementary Information Supplementary Table S7

Supplementary Information Supplementary Table S8

Supplementary Information Supplementary Table S9

Supplementary Information Supplementary Table S10

Supplementary Information Supplementary Table S11

Supplementary Information Supplementary Table S12

Supplementary Information Supplementary Table S13

## Figures and Tables

**Table 1:** CDS coverage of each platform

Platform	On-target region size*	CDS coverage predicted by probe design	Read bases of raw NGS data	Mean depth of CDS region [median]	CDS coverage of depth ≥10 called data	CDS coverage of depth ≥15 called data
Agilent SureSelect V4†	87.49 Mb	93.88%	6.84 Gb	79.15 [61.78]	90.57%	87.56%
Agilent SureSelect V5†	89.48 Mb	96.53%	6.38 Gb	73.10 [65.02]	93.85%	91.90%
NimbleGen SeqCap ez Human Library v2‡	81.58 Mb	96.08%	18.68 Gb	199.07 [172.00]	93.54%	92.98%
Illumina TruSeq‡	100.33 Mb	94.28%	11.40 Gb	61.72 [60.00]	90.23%	88.63%

*Within 100 bp upstream and downstream of the capture targets;

^†^SureSelect V4 and V5 data indicate average of 38 and 104 samples of our experiments, respectively;

^‡^the original data were obtained and have been deposited by Clark *et al.*^9^, and re-analyzed them with our analysis pipeline; More details are shown in Supplementary Table S1.

## Notes

**Accession code:** The compound heterozygous ASPM mutation has been deposited in NCBI ClinVar with accession number SCV000189126.

URLs. Exonic annotations from NCBI GRCh37.p10, ftp://ftp.ncbi.nih.gov/genomes/ MapView/Homo_sapiens/sequence/current/initial_release/seq_gene.md.gz; NimbleGen SeqCap EZ Human Exome Library annotation, http://www.nimblegen.com/products/seqcap/ez/index.html; Illumina TruSeq Exome Enrichment Kit annotation, https://support.illumina.com/sequencing/sequencing_kits/truseq_exome_enrichment_kit/downloads.ilmn; bedtools software, http://bedtools.readthedocs.org; NCBI SRA (obtained public WES data), http://www.ncbi.nlm.nih.gov/sra/; HaloPlex probe design software (SureDesign), https://earray.chem.agilent.com/suredesign/; BLAT software, http://hgdownload.cse.ucsc.edu/admin/exe/linux.x86_64.v287/blat; cutadapt software, https://code.google.com/p/cutadapt/; SAMtools software, http://samtools.sourceforge.net; GATK software, http://www.broadinstitute.org/gatk/; dbSNP, http://www.ncbi.nlm.nih.gov/SNP/; 1000 Genomes Project, http://www.1000genomes.org; ESP6500, http://evs.gs.washington.edu/ EVS/; HGVD, http://http://www.genome.med.kyoto-u.ac.jp/SnpDB/;HGMD, http://www.hgmd.org; ClinVar, https://www.ncbi.nlm.nih.gov/clinvar/; Information of ASPM protein domain in InterPro (v.47), http://www.ebi.ac.uk/interpro/protein/Q8IZT6; MyDomains tool for graphic depiction of protein domains, http://prosite.expasy.org/mydomains/.
