# Supplementary material for: A combination of targeted enrichment methodologies for whole-exome sequencing reveals novel pathogenic mutations
Source: Sci Rep. 2015 Mar 19;5:9331. doi: 10.1038/srep09331 (PMC4365396; doi:10.1038/srep09331)
Supplement: Supplementary Information [file srep09331-s1.pdf]

Supplementary Information

A combination of targeted enrichment methodologies for whole-exome sequencing reveals novel pathogenic mutations

Fuyuki Miya[1], Mitsuhiro Kato[2], Tadashi Shiohama[3], Nobuhiko Okamoto[4], Shinji Saitoh[5], Mami Yamasaki[6], Daichi Shigemizu[1], Tetsuo Abe[1], Takashi Morizono[1], Keith A. Boroevich[1], Kenjiro Kosaki[7], Yonehiro Kanemura[8,9] and Tatsuhiko Tsunoda[1]*

[1] Laboratory for Medical Science Mathematics, RIKEN Center for Integrative Medical Sciences, Yokohama, Japan.

[2] Department of Pediatrics, Yamagata University Faculty of Medicine, Yamagata, Japan.

[3] Department of Pediatrics, Graduate School of Medicine, Chiba University, Chiba, Japan.

[4] Department of Medical Genetics, Osaka Medical Center and Research Institute for Maternal and Child Health, Osaka, Japan.

[5] Department of Pediatrics and Neonatology, Nagoya City University Graduate School of Medical Sciences, Nagoya, Japan.

[6] Department of Pediatric Neurosurgery, Takatsuki General Hospital, Osaka, Japan.

[7] Center for Medical Genetics, Keio University School of Medicine, Tokyo, Japan.

[8] Division of Regenerative Medicine, Institute for Clinical Research, Osaka National Hospital, National Hospital Organization, Osaka , Japan.

[9] Department of Neurosurgery, Osaka National Hospital, National Hospital Organization, Osaka, Japan.

[*] Correspondence should be addressed to T.T. (tsunoda@src.riken.jp)

1. Supplementary Figure S1, S2 and S3	2 - 4
2. Supplementary Data 1 and 2	5 - 6

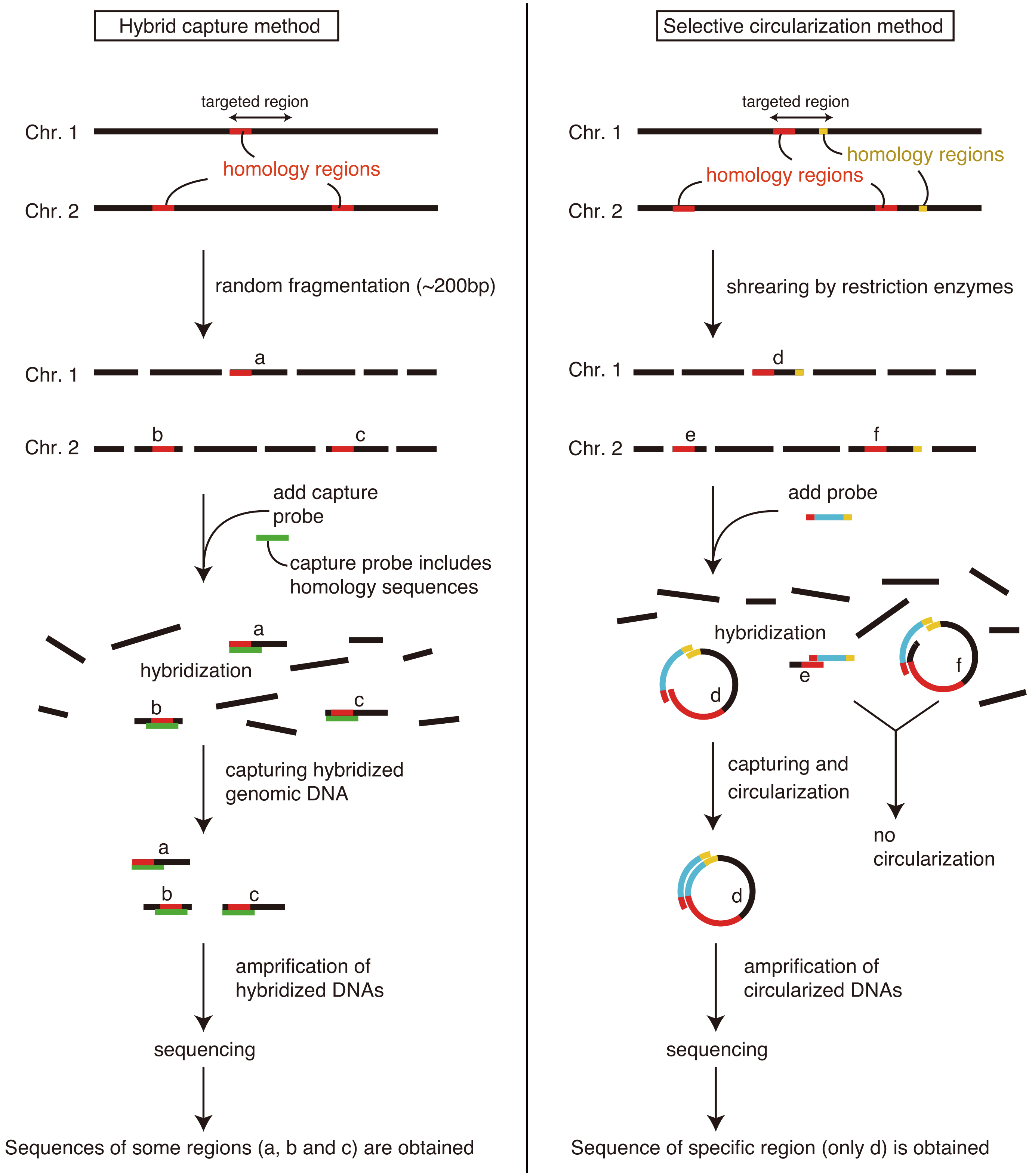

Supplmentary Figure S1. Advantage of selective circularization method compared to the hybrid capture method. Diagrammatic illustration indicates procedures of hybrid capture and selective circularization methods (many steps were abbreviated in this figure). In this case, a targeted region shares homology with other regions. The hybrid capture method tends to capture and sequence the homology regions together (a, b and c). The sequenced reads are mapped to some homologous regions in the reference genome, but the determining the original coordinates of the sequences is difficult. In contrast, the selective circularization method enables sequencing of single specific region (only d), because the selective circularization method is limited the circularization by strictly homologous with the probe and existence of restriction enzyme sites in both end of the targeted region. Although the sequenced read is also mapped to some homologous regions in the reference genome, almost all of non-original coordinates are feasibly rejected due to the potential of circularization.

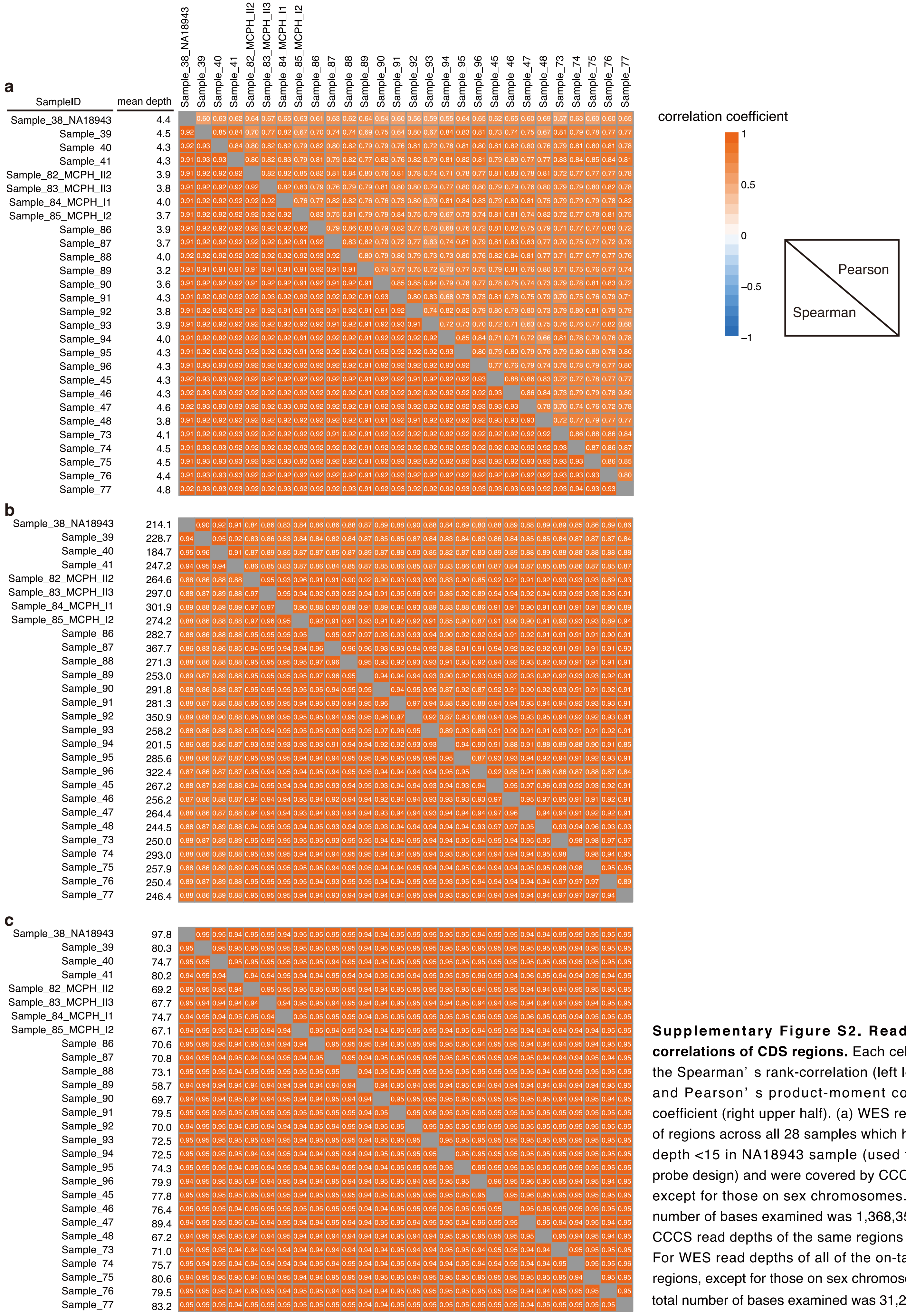

Supplementary Figure S2. Read depths correlations of CDS regions. Each cell contains the Spearman' s rank-correlation (left lower half) and Pearson' s product-moment correlation coefficient (right upper half). (a) WES read depths of regions across all 28 samples which had a read depth <15 in NA18943 sample (used for CCCS probe design) and were covered by CCCS probes, except for those on sex chromosomes. The total number of bases examined was 1,368,357. (b) For CCCS read depths of the same regions as (a). (c) For WES read depths of all of the on-target CDS regions, except for those on sex chromosomes. The total number of bases examined was 31,295,571.

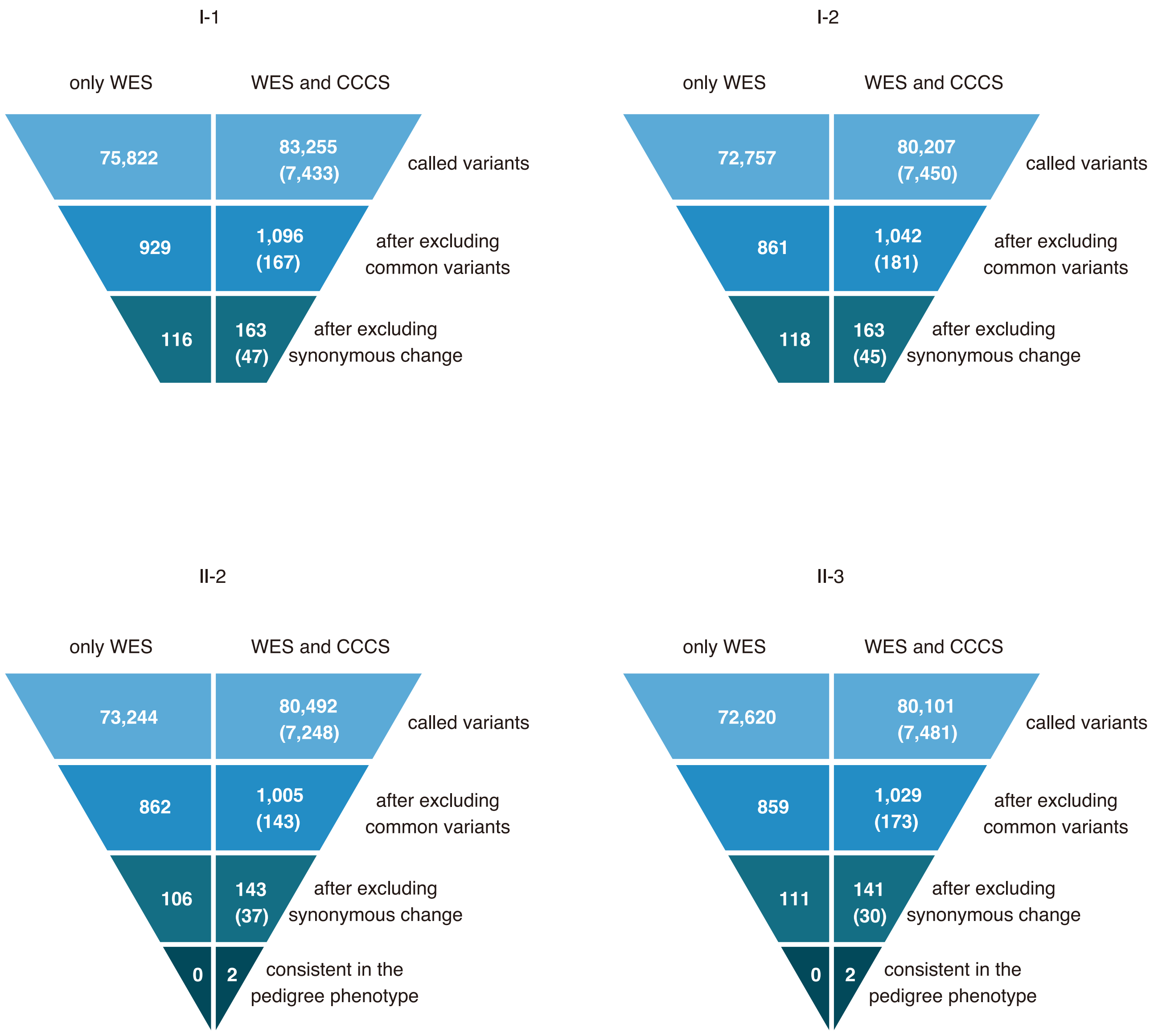

Supplementary Figure S3. Filtering candidate NGS mutations. Family tree of the pedigree with microcephaly is shown in Fig. 3a. The numbers in parenthesis represent the number of called variants with CCCS. Overlapping variants between WES and CCCS are not excluded. The top row shows the number of variants called by ‘WES’ and ‘WES and CCCS’ . The second row shows counts after excluding known variants found in databases, except for known pathogenic mutations. The third row shows variant counts after excluding synonymous changes. Finally, the last row of variant counts is consistent with the phenotype in the pedigree (i.e., total number of the autosomal recessive and compound heterozygous variants).

Supplementary Data 1. Evaluation of SNV calls.

To check the quality of our variant calling algorithm on whole-exome sequencing (WES) and custom complementary CDS sequencing (CCCS) data, we genotyped the 7 samples using Illumina HumanOmniExpressExome SNP array, and compared them with NGS data. Of the 7 genotyped samples, all 7 had WES data available, and 2 had CCCS data available. For WES, an average of 243,573 genotype locations were comparable between the two datasets and 99.95% genotypes were concordant between WES calls and the SNP array (Supplementary Table S7). We examined the 140 discordant calls for the NA18943 sample (SureSelectV4 WES data) using Sanger sequencing (Supplementary Table S8 and Supplementary Table S9) and 77 of 140 genotypes were consistent with our NGS calls. Based on this validation, we estimate the false positive and false negative rate of our variant calling method for WES to be 0.021% (50 / 243,573) and 0.064% (13 / 20,375), respectively. Note that these rates are conservative estimations as the SNVs that could not be successfully determined using Sanger sequencing were counted as incorrect. Moreover, the test sample, NA18943 SureSelectV4 data, had the most discordant SNP calls of the set. In the same manner, we compared the genotype calls between the CCCS and the SNP array, and 26,168 locations from 2 samples were comparable between the two datasets. Forty locations were discordant and the observed concordance rate was 99.85% (Supplementary Table S10). We examined the 40 locations genotype with Sanger sequencing and found 24 were consistent with our NGS calls (Supplementary Table S9 and Supplementary Table S11). Based on this validation, we estimate the false positive and false negative rate of our variant calling algorithm in CCCS calling to be 0.038% (10 / 26,168) and 0.27% (6 / 2,203), respectively.

Supplementary Data 2. Clinical symptoms of affected individuals with microcephaly with the compound heterozygous mutation identified in present study.

The family tree of the affected family with microcephaly is shown in Fig. 3a. The II-2 affected child was born by spontaneous delivery at 40 weeks of gestation after an uneventful pregnancy as the second child of healthy and non-consanguineous parents. Her birth weight was 2,228 g (−2.0 SD) and her HC was 29 cm (−3.2 SD). She showed no dysmorphism except for microcephaly (Fig. 3b). She showed mild delay of language development, though her intelligence and motor functions were normal at 6 years of age. Her HC was 42 cm (−6.6 SD) at 6 years of age. The affected child II-3 was a younger brother of II-2. He was born at 40 weeks of gestation with no asphyxia. His birth weight was 2,626 g and his HC was 29 cm (−3.2 SD). At the age of 3 years, his HC was 42 cm (−4.9 SD) and showed normal development. His physical and neurological findings were generally normal except microcephaly.
